# Improved Prediction of Regulatory Element Using Hybrid Abelian Complexity Features with DNA Sequences

**DOI:** 10.3390/ijms20071704

**Published:** 2019-04-05

**Authors:** Chengchao Wu, Jin Chen, Yunxia Liu, Xuehai Hu

**Affiliations:** 1 College of Informatics, Agricultural Bioinformatics Key Laboratory of Hubei Province, Huazhong Agricultural University, Wuhan 430070, China; ccwu@mail.hzau.edu.cn (C.W.); liuyunxia686@gmail.com (Y.L.); 2 College of Science, Huazhong Agricultural University, Wuhan 430070 China; cj@mail.hzau.edu.cn

**Keywords:** regulatory element, enhancer, abelian complexity, prediction

## Abstract

Deciphering the code of *cis*-regulatory element (CRE) is one of the core issues of current biology. As an important category of CRE, enhancers play crucial roles in gene transcriptional regulations in a distant manner. Further, the disruption of an enhancer can cause abnormal transcription and, thus, trigger human diseases, which means that its accurate identification is currently of broad interest. Here, we introduce an innovative concept, i.e., abelian complexity function (ACF), which is a more complex extension of the classic subword complexity function, for a new coding of DNA sequences. After feature selection by an upper bound estimation and integration with DNA composition features, we developed an enhancer prediction model with hybrid abelian complexity features (HACF). Compared with existing methods, HACF shows consistently superior performance on three sources of enhancer datasets. We tested the generalization ability of HACF by scanning human chromosome 22 to validate previously reported super-enhancers. Meanwhile, we identified novel candidate enhancers which have supports from enhancer-related ENCODE ChIP-seq signals. In summary, HACF improves current enhancer prediction and may be beneficial for further prioritization of functional noncoding variants.

## 1. Introduction

The development and cellular differentiation in eukaryotic organisms requires precise regulation of gene expression, which is governed by the orchestration of various *cis-*regulatory elements (CRE) [1]. Enhancer is one category of CRE that regulates the transcription of target gene in a distal manner [2,3,4]. On the one hand, the ENCODE project identified >500,000 putative enhancers using histone modifications indirectly [5,6] and their total length might reach 12% of the human genome [7]. On the other hand, genome-wide association studies (GWAS) in the past decade identified over 55% of the disease-associated SNPs in the non-coding DNA [8] and some of them were located exactly in the enhancer regions [9]. Therefore, accurate identification of enhancer region across the whole genome is currently of broad interest. It is urgent that we systematically discover active enhancers in specific cells or tissues for a better understanding of the functional roles of non-coding variants.

However, this is a challenging problem because an enhancer acts in its regulating role with both a distal and bidirectional manner. In contrast to promoter, distal enhancers are typically located more than 10 kb away from the gene it regulates [4], and some extreme examples showed that the distance might be farther away, such as 1 Mb [10,11]. Such a long distance makes precise positionings of enhancers difficult. Also, enhancers perform diverse regulatory functions during different tissues and cell lines. For instance, the genomic region of chr11:110,716,521–110,832,306 in diffuse large B-cell lymphoma (DLBCL) acts as an enhancer that specifically regulates POU2AF1 [12], and the genomic region of chr22:27,552,905–27,512,763 acts as an enhancer with activating expression of XBP1 in the multiple myeloma cell line (MM1.S) [13]. The specificities of enhancers among different tissue and cell lines make it harder to discover novel enhancers. Moreover, unlike a promoter that is located in the upstream of its target gene, an enhancer can function bidirectionally, which doubles the searching difficulty [3].

In the past two decades, researchers developed several distinct experimental strategies from different viewpoints for inferring the locations of active enhancers, such as Vista enhancer browser using transgenic mouse assay [14], EP300 (histone acetyltransferase, an ubiquitous activator) enhancer using ChIP-seq of EP300 [15], ENCODE enhancer inferred from chromatin features of ENCODE data [5,6,16,17,18], experimental-validated enhancer by massively parallel report assay (MPRA) [18,19,20], STARR-seq using self-transcribing transcripts [21] and FANTOM enhancer identified with cap analysis of gene expression (CAGE) of enhancer RNA (eRNA) [22]. Among them, VISTA enhancer browser [14] used a transgenic mouse assay to experimentally validate activities of candidate enhancers (human or mouse noncoding fragments supported with their extreme conservation or putative enhancer markers) *in vivo*. ChromHMM [5] or Segway [6] adopted an unsupervised clustering approach for annotating genome-wide enhancers based on a variety of chromatin features (such as chromatin accessibility with DNase I Hypersensitive Sites (DHSs), covalent histone modifications including monomethylation of lysine 4 of histone H3 (H3K4me1) and acetylation of lysine 27 of histone H3 (H3K27ac) from ENCODE data. However, these annotations are not satisfactory since an example of a study using MPRA demonstrated that only around 20% of ENCODE annotated enhancers in K562 cell lines drove significant activity of a reporter gene [20]. More recently, enhancers were found to be acting as transcription units and to be producing eRNA in a widespread manner [23]. Based on this, the FANTOM consortium [22] employed CAGE to analyze eRNA peaks around enhancers and found a universal pattern that enhancer exhibits a bidirectional transcription both in the forward and reverse strands. Taking this as a criterion, they identified the exact location of enhancer to be the interval between the two CAGE peaks (FANTOM5 enhancer data). Although a golden criterion for accurate enhancer identification is still obscure, nowadays the strategy employing eRNA has received increasing acceptances [3,24,25] and FANTOM5 enhancer data has become the state-of-the-art data resource for discovering enhancers [1].

Another possible strategy for enhancer identification is by a computational method from the DNA sequence only. The rationale behind this lies in two aspects: (1) enhancer sequence contains combinations of short sequence motifs known as transcriptional factor binding sites (TFBSs) and disrupting their core motifs substantially reduced the activities of the enhancers [26], suggesting that these motif features are necessary for enhancer activity; (2) even when being removed from its endogenous context and moved to upstream of a reporter gene, enhancer sequence can still drive gene expressions [27], suggesting that enhancer DNA is sufficient for enhancer activity [28]. The above two points make the sequence-based computational mean an alternative strategy for enhancer identification.

The computational strategy aims to learn *cis*-regulatory code (CRC) [29] hidden in trustable enhancer samples and then to build a reliable prediction model both for evaluating existing enhancers and for discovering novel enhancers [26,30,31,32,33,34]. A pioneer finding is that *k-mer* features of varying length (3–10 bp) (an optimal *k* is 6) are predictive sequence features for discriminative prediction of enhancer when analyzing ChIP-seq data of EP300 [30]. Notably, a motif discovery of STARR-seq enhancer data found that known TF motifs as well as dinucleotide repeat motifs (DRMs) are enriched in enhancer sequences [26]. Further, a support vector machine (SVM) model using these differential motif distributions was sufficient (AUC = 0.90) to discriminate enhancers from negative controls. Moreover, DEEP integrates three resources of enhancer data, ENCODE, FANTOM5 and VISTA to develop an ensemble prediction framework by using 351 attributes derived from the sequences including *1–4 mers* and other 11 combinatoric features [31]. iEnhancer-2L proposed to use pseudo *k-tuple* nucleotide composition features for identifying enhancers and their strength [32]. More recently, more advanced tools that can automatically learn CRC, including deep learning [33] and gkm-SVM [34], were applied for predicting enhancers.

Although different tools adopted distinct technologies, a consensus is that one should try to learn combinatoric properties of DNA motifs or *k-mer* to be predictive sequence features. Notably, DRMs like “GCGCGC” and “GAGAGA” were proven to be necessarily required for active enhancers and a high occurrence number of DRM was found to be a main feature of broadly active enhancers [26]. In mathematics, subword complexity function (SCF) and topological entropy (TE) characterize complexity of DNA sequence by counting the number of different *k-mers* (termed as “subwords” in mathematics) appearing in the DNA sequence. This enables SCF or TE to be the most appropriate tools for characterizing repeat sequence [35] because repeat sequence like “GCGCGC” uses less subwords (only two distinct subwords with length 2–5). Naturally, we tried to apply TE for discriminating enhancers from other types of DNA elements. As expected, TE is sufficient for discriminating enhancers from promoters. Unexpectedly, when discriminating enhancers from non-enhancers, TE is no longer efficient, suggesting that a new method is required. Here, we introduce a novel sequence complexity concept, i.e., abelian complexity function (ACF), for a new coding of DNA sequences. After feature selection supported with an upper bound theorem and integration with *4-mer* motif features, hybrid abelian complexity features (HACF) are proven to be predictive for enhancer prediction.

## 2. Results

### 2.1. Topological Entropy of Enhancer is Larger than that of Exon, Intron or Promoter

**Figure 1:**
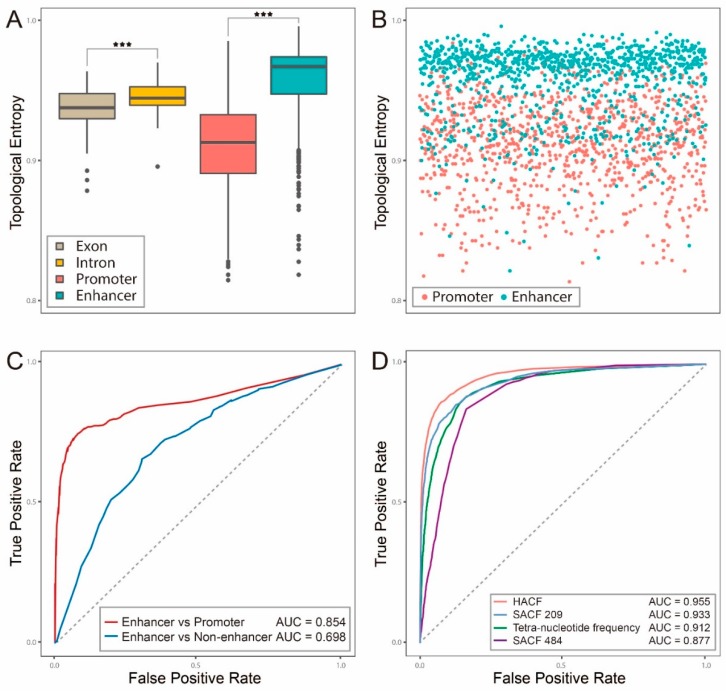
Topological entropies (TEs) of four distinct genomic elements and discriminating enhancer from promoter or non-enhancer with TE. (**A**).Box-plots of TEs of four distinct genomic elements including intron, exon, promoter and enhancer; *** represents *p*-value < 0.001; (**B**)The point-plot of TEs of 200 longest promoters (red) against 200 longest enhancers (green); (**C**) Two ROC curves of prediction performances of two models with TE: enhancer versus promoter (red) and enhancer versus non-enhancer (blue); The p-value of T test between the AUC of two groups is 1.4 × 10^−7^; (**D**) ROC curves of prediction performances of four feature inputs on the dataset Enhancer4589. HACF: hybrid abelian complexity features; SACF484: the 484 selected abelian complexity features determined by an upper bound estimation; SACF209: the 209 selected abelian complexity features determined by Wilcoxon-test; The p-value of T test between AUC of HACF and SACF209 is 8.1 × 10^−3^; the p-value of T test between AUC of SACF209 and tetra-nucleotide frequency is 2.5 × 10^−3^; the p-value of T test between AUC of tetra-nucleotide frequency and SACF484 is 4.9 × 10^−5^.

Different functional genomic regulatory elements have distinct TEs: it was reported that *H_top_*(intron) > *H_top_*(exon) > *H_top_*(promoter)(*H_top_* denotes the topological entropy) [36,37], leading us to naturally ask about TE of enhancer. Toward this end, we applied the formula (4) in corollary 1 to compute TEs of four categories of genomic elements: exon, intron, promoter and enhancer. To be similar to a previous study which employed TE for discriminating intron and exon [36], we chose the longest 200 introns and exons, respectively, based on the GRCh37 reference genome. For promoter and enhancer data, we chose the longest 200 promoters from FANTOM5 promoter data [38] and the longest 200 enhancers from the dataset Enhancer4589, respectively. To be consistent with previous findings, the relationship of *H_top_*(intron) > *H_top_*(exon) > *H_top_*(promoter)still holds. And, as expected, *H_top_*(enhancer) is significantly larger than that of exon, intron or promoter (Figure 1A), suggesting that enhancer sequence uses more distinct subwords and is more random than other genomic elements.

It is noteworthy that the difference of TE between promoter and enhancer is large (Figure 1B), leading us to ask whether we could distinguish enhancer from promoter with TE. We then ran a SVM classifier on the above longest 200 enhancers (positives) and the longest 200 promoters (negatives), and the resulting AUC was 0.854 (Figure 1C), suggesting that TE is a good predictor for discriminating enhancer from promoter. However, when the negatives were replaced to random intergenic regions without any annotations, the resulting AUC dropped down to 0.698 (Figure 1C), suggesting that TE is not sufficient for predicting enhancer in the genome-wide scale and that a new method is required.

### 2.2. Hybrid Abelian Complexity Features are Predictive Features for Enhancers

**Figure 2:**
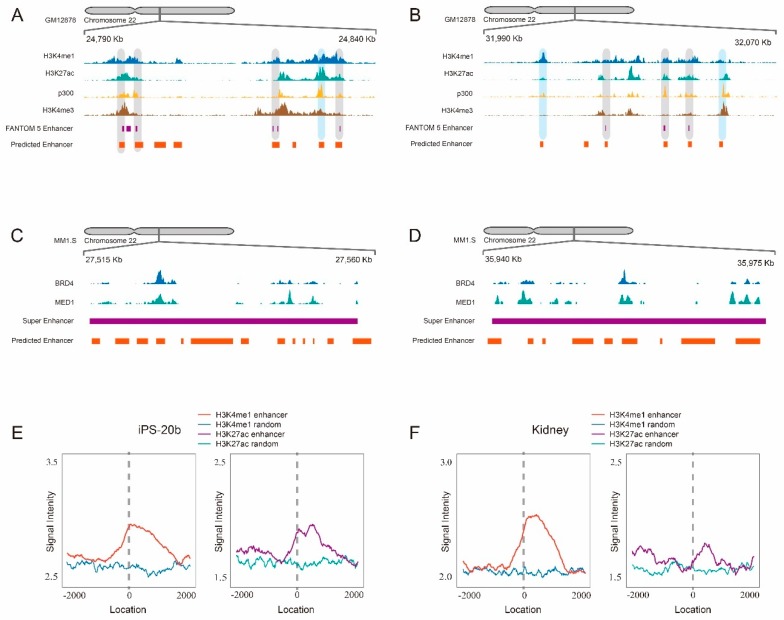
Enhancer-related ENCODE ChIP-seq signals supports the predicted enhancers to be candidate enhancers. (**A**) A representative region within human chromosome 22 for showing that the predicted enhancers have supports either from FANTOM5 enhancer data (shadow regions with gray color) or from ENCODE ChIP-seq signals including H3K4me1, H3K27ac, EP300 and H3K4me3 of GM12878 cell line (shadow regions with blue color); (**B**) Another representative region; (**C**) A case study of a 3.9 kb super-enhancer (chr22:27516134–27555928) which is related to the multiple myeloma in MM1.S cell line;.(**D**) Another case study of a 3.1 kb super-enhancer (chr22:35940694–35972007); (**E**) Average signal intensities of two ENCODE ChIP-seq datasets (H3K3me1, red curves and H3K27ac, purple curves) of iPS-20b cell line on the predicted enhancer regions (± 2Kbcentered at the predicted enhancers) versus the random genomic regions (blue and green curves); (**F**) A similar demonstration in the adult kidney tissue.

Next, we proposed a new concept of sequence complexity—abelian complexity to construct a prediction model of enhancer based on the dataset of Enhancer4589. We first applied SACF484 (Methods 2.4) to run a SVM classifier and found a predicting accuracy of AUC of 0.877 (Figure 1D and Table 1). We further selected 209 abelian complexity features (SACF209, Supplementary Table S2) among 484 features by a statistical test between positive and negative samples (Wilcoxon-test, p-value <1.0 × 10^−20^, light blue points in Figure 2C). When using SACF209 for prediction, a significant increase was achieved with AUC of 0.933 (Figure 1D and Table 1).

DNA composition features are fundamental features for representing DNA sequences and tetra-nucleotide frequency features (TNF, 256 4-*mer* features by counting frequencies of four continuous bases) were proven as predictive features for identifying enhancers in DEEP [31]. Finally, when integrating SACF209 and TNF to construct a combined feature set with 465 features in total (hybrid abelian complexity features, HACF), the predicting accuracy further achieved a slight promotion with AUC of 0.955 (Table 1). Notably, when comparing SACF209 with TNF, AUC of 0.933 of the former is better than AUC of 0.912 of the later. Moreover, we ran a feature importance program to rank all the 465 features by each feature’s contribution to prediction and found that there are 88 features out of SACF209 (dark blue points in Figure 2D, Supplementary Table S3) among the top 100 important features. These findings all support the conclusion that SACF209 are predictive features and HACF is a more optimal combined feature set for enhancer prediction.

### 2.3. Comparisons with Other Existing Methods

To comprehensively compare with existing methods, we chose three state-of-the-art models, EnhancerFinder [39], DEEP [31] and iEnhancer-2L [32] for comparisons. EnhancerFinder constructed a dataset including 717 VISTA human enhancer sequences and 717 random genomic regions, however it did not provide the dataset in their publication [39]. We turned to the latest version of the VISTA database (February 08, 2018) and downloaded 959 human enhancer sequences and we randomly chose 959 intergenic regions with the same length distribution of positive samples to construct the negative sample set. When running our model (HACF) on the newly-constructed VISTA enhancer dataset using 10-fold cross validation, the AUC of 0.962 outperformed the AUC of 0.960 of EnhancerFinder (Table 2).

DEEP [31] performed tissue-specific enhancer predictions based on FANTOM5 enhancer data, however it only provided the detailed prediction results on three specific tissues: heart, liver and brain, which were chosen for comparisons. The same enhancer data of three tissues of DEEP was used as positive samples and the negative samples were chosen from random intergenic regions with 10 times the number of positive samples of each tissue (Supplementary Table S4). When performing the optimal testing strategy (40% for training and 60% for testing) of DEEP, the ACC and AUC values of HACF outperform those of DEEP while the GM values are relatively lower (Table 2). This suggests that HACF is generally comparable with DEEP. On the one hand, prediction performance of HACF on some indexes like AUC are superior to those of DEEP. On the other hand, DEEP has greater GM values and is more balanced between positive predictions and negative predictions.

iEnhancer-2L [32] constructed a two-layer predictor where 742 strong enhancers and 742 weak enhancers annotated by ChromHMM [5] were combined as positive samples in the first layer (1484 non-enhancers as negative samples). And the second layer was further designed to distinguish 742 strong enhancers and 742 weak enhancers. After downloading the provided dataset of iEnhancer-2L (http://bioinformatics.hitsz.edu.cn/iEnhancer-2L/data/) (access on 5 May 2018), we ran the HACF model and listed a detailed comparison based on a leave-one-out test in Table 2. An AUC of 0.907 of the first layer and an AUC of 0.789 of the second layer outperform 0.850 and 0.660 of iEnhancer-2L, respectively, suggesting that HACF is superior to iEnhancer-2L in both layers.

In summary, comprehensive comparisons with three state-of-the-art methods might lead us to conclude that our HACF approach is a more accurate and robust model for enhancer prediction.

### 2.4. Scanning Chromosome 22 Identifies Known Disease-Related Super-Enhancers as Well as Novel Candidate Enhancers

Finally, we applied the HACF model to scan the whole DNA sequence of human chromosome 22 for validating its generalization ability. We adopted a scanning strategy by sliding a 1 kb-window with step size 10 bp across human chromosome 22 (34,885,300 bp in total), by which 3,488,530 DNA fragments were obtained and then were put into the HACF model for prediction. To control the false positive, we adopted a similar analysis strategy with ChIP-seq for the quality-control task: (1) each 1 kb DNA fragment that had a predicted value larger than 0.5 was regarded as a candidate read; (2) all candidate reads along with their predicted values were put into MACS2 [41] to perform peak calling. As per the output, MACS2 gave 7045 peaks that were considered as the predicted enhancers (Supplementary Table S5). As a result, 869 out of 1151 (75.5%) FANTOM5 enhancers within chromosome 22 are consistent with our predicted enhancers (with overlap), which further validated our model. For example, we listed two representative regions validated in GM12878 cell lines (Figure 2A,B) where the regions that are covered by gray color represent the overlapping enhancers with FANTOM5 enhancers. Notably, these regions are also supported with neighboring enhancer-related epigenomic markers of H3K4me1, H3K27ac, EP300 and H3K4me3 (Figure 2A,B), suggesting that the HACF model is an accurate model for enhancer identification.

Meanwhile, we tested the generalization ability of the HACF model on two known super-enhancers (SE) located within chromosome 22 which were reported to be related with MYC oncogene in multiple myeloma [13]. Lovén et al. used the ChIP-seq signals of coactivator BRD4 and MED1 to identify the locations of SE and showed that SE are key oncogenic drivers in many tumor cells [13]. They totally identified 308 SE of the multiple myeloma cell line (MM1.S), six of which are located within chromosome 22. We chose two of them (chr22:27516134-27555928, 3.9 kb and chr22:35940694-35972007, 3.1 kb) to perform two case studies. We used the same strategy above to scan these two regions and identified enhancer peaks (Figure 2C,D), most of which are quite consistent with ChIP-seq peaks of BRD4 and MED1, suggesting that the HACF model has a powerful ability of identifying disease-related super-enhancers.

In addition, our scanning approach simultaneously identified some novel candidate enhancers, some of which are covered by a blue color (Figure 2A,B), representing those candidate enhancers that have no overlaps with FANTOM5 enhancers yet are supported by at least one of ChIP-seq peaks of H3K4me1, H3K27ac, EP300 and H3K4me3 markers (downloaded from https://www.ncbi.nlm.nih.gov/geo/roadmap/epigenomics/) access on June 2, 2018. To comprehensively evaluate validities of novel candidate enhancers, we made comparisons between 7045 candidate enhancer regions (±2Kbcentered at the predicted enhancers) and the same number of random intergenic regions (background) on their average signal intensities of H3K4me1 and H3K27ac in two cell lines or tissues (iPS-20b and kidney). It was found that both H3K4me1 (red curves) and H3K27ac (purple curves) signals on candidate enhancer regions generated obvious peaks around the locations of predicted enhancers which are significantly larger than the signals on the random regions (blue and green curves) (Figure 2E,F), supporting that the enhancers predicted by the HACF model have the potential to be bona fide enhancers.

## 3. Methods

### 3.1. Dataset Preparation

In this study, we constructed a large-scale enhancer dataset based on FANTOM5 enhancer data [22], which can be downloaded at: http://fantom.gsc.riken.jp/5/datafiles/latest/extra/Enhancers/ (access on January 11, 2018). The original data was presented as a matrix *E*_65423×1829_ with 65,423 rows and 1829 columns, where each row represents a specific enhancer, each column represents a specific tissue or cell line in human and element in *ith* row and *jth* column e*_ij_* epresents the relative log expression (RLE)-normalized expression value (presented as tags per million mapped reads, TPM) of CAGE of *ith* enhancer in *jth* tissue or cell line.

To construct a high-quality enhancer dataset, the following four major steps of data preprocessing were involved. First, Andersson et al. [22] found that bidirectional capped RNA is a signature feature of active enhancers, which implies that larger TPM ensures greater active intensity of enhancer. Using this principle, we adopted a cut-off criterion of ‘*TPM*_rowmin_ ≥ *α*’ (*TPM*_rowmin_ represents the minimal nonzero value of TPM across all tissues and cell lines of a given enhancer, *α* is a given positive parameter) to select most active enhancers. When *α* was determined as 0.08 (we showed that the prediction performance was not sensitively dependent on the selection of parameter *α* in the supplementary information and *α* = 0.08 is an appropriate choice), only 5386 enhancers passed this criterion. Next, although some existing works used 2 kb as the length of enhancer sequence [42,43], we fixed all the input sequences to a length of 1 kb because FANTOM enhancer sequence has a shorter length (282 bp on average) compared with ENCODE enhancer sequence. Since a previous study reported that too short a sequence would lead to failed feature extraction [44], we therefore excluded those enhancers with lengths shorter than 100 bp and obtained 4667 enhancers. Meanwhile, for sequences that were larger or shorter than 1 kb, they were truncated at the 5’ and 3’ ends or were expanded to 1 kb based on the human reference genome (hg19), respectively, centering at the middle points. In the third step, we employed a redundancy reduction procedure CD-HIT [45] (http://www.bioinformatics.org/cd-hit/) (access on February 12, 2018). with a cutoff threshold of 0.8 to remove the redundancy between enhancer sequences, meaning 4653 enhancers remained. In the last step, for a fair evaluation of scanning chromosome 22, we discarded those enhancers located within chromosome 22 and obtained a total of 4589 enhancers as the final positive sample dataset for training (Enhancer4589, Supplementary Table S1).

To help the discrimination of enhancers, a negative sample dataset is required. Recent studies reached a consensus of using ten times the number [31,32,33] or seven times the number [43] of positives from promoter regions or randomly chosen intergenic regions to construct the negatives. The rationale behind this lies in that enhancers account for around 12% of the human genome [7] and the training model should adopt a similar ratio (1:10 or 1:7) of positives and negatives, otherwise the trained model will predict a large number of false-positives when scanning genome, resulting in an invalid prediction model. Therefore, we then randomly selected 10 times (45,890) the amount of 1 kb DNA sequences as negative samples (Enhancer4589, Supplementary Table S1) from non-enhancer intergenic regions, which are defined 1 kb regions based on the GRCh37 reference genome by excluding exon, intron and known enhancers.

### 3.2. Subword Complexity Function and Topological Entropy

Subword complexity function (SCF) studies combinatorial features of sequence by counting the number of different subwords appearing in the sequence and topological entropy (TE) is a mathematical index for quantitatively measuring the asymptotic exponential increasing speed of SCF [35]. They were originally proposed to study problems in the areas of natural language processing, pattern matching and coding theory [35], and were transferred to study DNA sequences in the past two decades [36,46,47,48,49]. More precisely, let Ω = {*A,C,G,T*} be an alphabet with four letters, the SCF of a given DNA sequence *ω* over Ω with a finite length |*ω*| is defined as:

**Definition** **1.** *(Subword Complexity Function, SCF [35,36,49]) For a DNA sequence ω over Ω, the subword complexity function p_ω_: N→N is given by*

(1) $$p_{\omega }\left(n\right)=Card\left\{\mu :\mu \to \omega ,\left|\mu \right|=n\right\}$$

*where Card denotes the number of elements of a set and μ < ω represents that μ is a subword (or a factor) of ω.*

Mathematically, the nature of 1 ≤ *p_ω_*(*n*) ≤ 4*^n^* holds for an arbitrary DNA sequence, and *p_ω_*(*n*) = 1 represents a complete repeat sequence, whereas *p_ω_*(*n*) = 4*^n^* represents the sequence containing all subwords with full complexity. In early works, Kirillova [47] and Colosimo and De Luca [46] studied DNA sequences by using the entire SCF, which suffers a high-dimensionality problem when the studied sequence is very long [36]. To grab the most significant complexity features, Koslicki [36] noted that the SCF is roughly strictly increasing, nondecreasing and linear decreasing with a slope of -1 on three disjoint intervals, the third of which carries no information. For the first two parts, Koslicki tried to use a unique index, i.e., TE, to quantitatively measure the complexity of a DNA sequence. The classic definition of TE of infinite *ω* over Ω is defined as:

**Definition** **2.** *(Topological Entropy, TE [35]) For an infinite sequence ω over Ω, the topological entropy H_to_ is defined by*

(2) $$H_{top}\left(\omega \right)=\underset{n\to \infty }{\lim}\frac{log_{4}p_{\omega }\left(n\right)}{n}$$

The direct computing of TE is difficult because the classic definition of TE is based on infinite sequences equipped with the limit operation, which requires us to give an asymptotic algorithm to estimate the value of TE. A rational estimation of TE of *ω* was computed at the maximal value of *n* such that 4*^n^* + n – 1 ≤ |*ω*| based on the following theorem and corollary [36]:

**Theorem** **1.** *(Existence of sequence with full complexity [50]) For all n ≥ 0 there exists a word ω over Ω with |ω| = 4^n^ + n – 1 such that*

(3) $$p_{\omega }\left(i\right)=4^{i},\;i=1,\ldots ,n.$$

**Corollary** **1.** *(Koslicki’s algorithm of TE [36]) Let ω be a finite sequence of length |ω|, and let n_0_ be the unique integer such that4^n^_0_ + n_0_ – 1 < |ω| ≤ 4^n^_0_^+ 1^ + (n_0_ + 1) − 1, then*

(4) $$H_{top}\left(\omega \right)\approx \frac{log_{4}p_{\omega }\left(n_{0}\right)}{n_{0}}.$$

Using the above algorithm, Koslicki gave a unique value of TE at *n*_0_ instead of the entire SCF for measuring the complexity of a DNA sequence. By applying this to the intron and exon elements of the human genome, he found that the complexities of introns are larger than those of exons, suggesting that intron is more random and is under less selective pressure. After that, Jin et al. [37] found that neighbouring points around *n*_0_ should be integrated together to give a more accurate estimation of TE. By applying it to three categories of genomic elements, they found that intron > exon > promoter of their complexities. Recently, Wu et al. [49] found that the neighbouring complexity features around *n*_0_ are good predictors for predicting DNA methylation. These related works altogether demonstrate that specific positions of SCF where it may reach full complexities are intrinsic features as well as good predictors for discriminating distinct functional genomic elements.

### 3.3. Abelian Complexity Function and Its computing Resource Consumption

Recently, Richomme et al. [40] comprehensively studied the general theory of abelian complexity function (ACF) and emphasized the importance of characterizing sequences by ACF and SCF simultaneously. Here, we introduce the concept of ACF to study DNA sequences:

**Definition** **3.** *(Parikh vector, PV [40]) For a finite sequence ω over Ω, the Parikh vector of ω is defined as the functionψ: Ω^|ω|^→N^4^, given by*

(5) $$\psi \left(\omega \right)=\left({\left|\omega \right|}_{A},{\left|\omega \right|}_{C},{\left|\omega \right|}_{G},{\left|\omega \right|}_{T}\right),$$

*where |ω|_A_ denotes the number of occurrences of the letter A ϵ Ω in the finite sequence ω.*

**Definition** **4.** *(Abelian Complexity Function, ACF [40]) For a finite sequence ω over Ω, the abelian complexity function ρ^ab^: N→N is defined as:*

(6) $$\rho_{\omega }^{ab}\left(n\right)=Card\left\{\psi \left(\mu \right):\mu \to \omega ,\left|\mu \right|=n\right\},$$

*where ψ(ω) is the Parikh vector of subword u of the finite sequence ω.*

**Figure 3:**
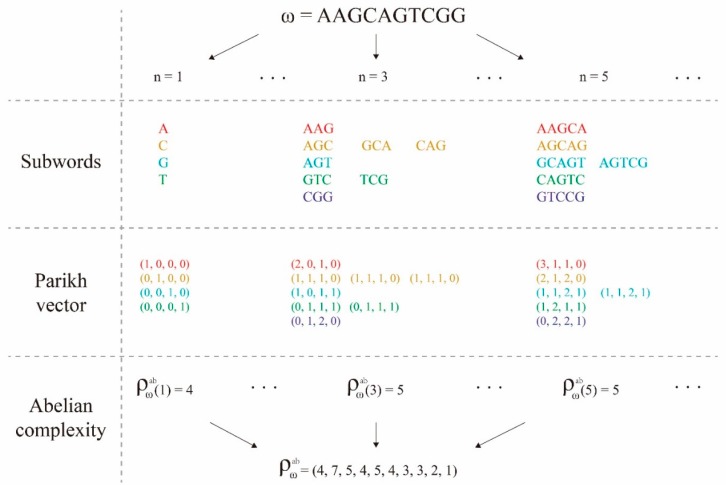
An example for showing the computational algorithm of abelian complexity function.

Note that for the finite sequence *ω*, if *n* > |*ω*|, then $\rho_{\omega }^{ab}\left(n\right)=0$. We always assume that *n* < |*ω*| when computing $\rho_{\omega }^{ab}\left(n\right)=0$. For better understanding of ACF, we showed the detailed computing process of it of an example of a given DNA fragment in Figure 3, from which we could find that ACF only counts the number of different PVs, differing from SCF which counts the number of all different subwords. This means $1\leq \rho_{\omega }^{ab}\left(n\right)\leq \rho_{\omega }\left(n\right)$ for all *n* ≥ 1. Another marked difference between ACF and SCF was that ACF usually showed a fluctuation while SCF strictly increased in the first part. For example, for the given DNA fragment *ω*=*AAGCAGTCGG* used in Figure 3, its ACF is, from which we could find that $\rho_{\omega }^{ab}\left(2\right)=7>5=\rho_{\omega }^{ab}\left(3\right)$. We drew a graph of ACF of an enhancer sequence with 1 kb in Figure 2A, which helps us to understand the fluctuation property of ACF.

We next investigated the resource consumption of computation of ACF by running our script on a test computer with Ubuntu 18.04 on Intel Xeon 2.10 GHz processors and 256 GB RAM. When running on 500 enhancer sequences with a length of 1 kb, a total of 1319.767 s and 3656.48 Mb were needed for such computations, implying that the average computation time and memory consumption of each DNA sequence was about 2.64 s and 7.313 Mb, respectively.

### 3.4. Upper Bound Theorem and Feature Selection

For a long DNA sequence, we now face the same problem of high-dimensionality as with the subword complexity situation. The main experience learnt from previous studies [36,37,49] is that we should select those positions where ACF might reach its maximums (full complexity). Therefore, we are required to give the upper bound estimation of ACF by the following theorem:

**Theorem** **2.** *(Upper bound of ACF) For every finite word ω and every positive integer satisfying 1 ≤ n ≤ |ω|, the absolute upper bound of ACF is given by*

(7) $$1\leq \rho_{\omega }^{ab}\left(n\right)\leq C_{n+3}^{3}=\frac{\left(n+3\right)\left(n+2\right)\left(n+1\right)}{6}$$

*where C means the binomial coefficient. Proof. We gave the proof of theorem 2 in the supplementary information.*

Under the guidance of the main idea of computing TE, we employed the upper bound of ACF in theorem 2 to estimate the minimal length required for reaching full abelian complexity. When determining the most appropriate position for computing TE, one chose the largest integer *n*_0_ such that 4*^n^_0_* + *n* – 1 ≤ |*ω*| because the minimal length required for reaching full subword complexity 4*^n^_0_* is 4*^n^_0_* + *n* – 1 (theorem 1). Here, we determine such a length for full abelian complexity by the following theorem:

**Theorem** **3.** *(Minimal length for reaching full abelian complexity) For all m ≥ 0, if there exists a word ω such that*$\rho_{\omega }^{ab}\left(i\right)=C_{i+3}^{3},i=1,\ldots ,m$*, then*$\left|\omega \right|\geq C_{i+3}^{3}+m-1$.

**Proof.**  Suppose there exists a word *ω* with full abelian complexity, which means $\rho_{\omega }^{ab}\left(i\right)=C_{i+3}^{3},i=1,\ldots ,m$ (such word must exist because full subword complexity guarantees full abelian complexity). Then it must have $\rho_{\omega }^{ab}\left(m\right)=C_{m+3}^{3}$. Therefore, there must exist a subword μ* of *ω* such that it has at least $C_{m+3}^{3}$ different factors, which requires that μ* at least has the length of $C_{m+3}^{3}+m-1$ to place all the different factors. This immediately leads

$$C_{m+3}^{3}+m-1\leq \left|\mu^{*}\right|\leq \left|\omega \right|.$$

 □

**Figure 4:**
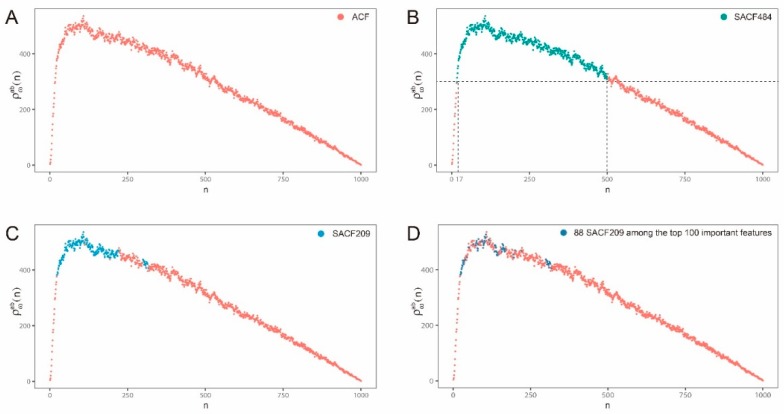
The graph of abelian complexity function and three groups of selected abelian complexity features. (**A**) The original graph of abelian complexity function (ACF); (**B**) The 484 selected abelian complexity features (SACF484, dark green) determined by an upper bound estimation; (**C**) The 209 selected abelian complexity features (SACF209, blue) further determined by Wilcoxon-test; (**D**) The 88 abelian complexity features (dark blue) among the top 100 important features.

Based on our experience of determining intrinsic features, we should focus on the middle region with high abelian complexity by discarding both ends of the ACF. For the head end, based on the above theorem 3, if |*ω*| = 1000, we determined the integer *m*_0_ = 16 such that $1000=\left|\omega \right|\geq C_{m_{0}+3}^{3}+m_{0}-1$. The abelian complexity features that are smaller than 17 were considered as irrelevant features that were discarded in the further studies. For the back end, we discarded the back-end region which had abelian complexities smaller than $\rho_{\omega }^{ab}\left(17\right)$ (under the dashed line in Figure 4B). The selected abelian complexity features are $\{\rho_{\omega }^{ab}(i),\;17\;\leq \;i\;\leq \;500\}$ (484 features, denoted by SACF484) in the case of |*ω*|=1000 (Figure 4B).

### 3.5. Evaluation of the Prediction Performance

Generally, the performance of a prediction method is measured by sensitivity (Sens or recall), specificity (Spec), precision, accuracy (ACC), Matthew’s correlation coefficient (MCC) value and GM value, which are calculated as:

(8) $$\{\begin{array}{c} Sens=recall=\frac{TP}{TP+FN},Spec=\frac{TN}{TN+FP},precision=\frac{TP}{TP+FP}\\ ACC=\frac{TP+TN}{TP+FP+TN+FN},GM=\sqrt{precision*recall}\\ MCC=\frac{TP*TN-FP*FN}{\sqrt{\left(TP+FN\right)*\left(TP+FP\right)*\left(TN+FP\right)*\left(TN+FN\right)}} \end{array}$$

where TP represents the number of true positive (enhancers predicted as enhancers), TN represents the number of true negative (non-enhancers predicted as non-enhancers), FP represents the number of false positive (non-enhancers predicted as enhancers) and FN represents the number of false negative (enhancers predicted as non-enhancers). Additionally, to test the balance between true positive and false positive rates, another evaluating index is the Area Under the ROC Curve (AUC).

## 4. Discussion

An enhancer is one class of the most important CRE and plays significant roles in the regulation of gene expressions. Identification of the locations of enhancers is currently of broad interest because it is a prerequisite for a better understanding of complex mechanisms of transcriptional regulations and for evaluating non-coding DNA variants. Subword complexity function and topological entropy were proven useful tools for discriminating distinct classes of CREs, such as intron, exon and promoter. However, it is no longer valid for the identification of enhancers, which is why we developed a new mathematical tool termed “abelian complexity function” to try achieve this task. Under an estimation of the upper bound, a feature selection approach finally determines 209 abelian complexity features that outperformed three existing methods in fair comparisons. Moreover, in addition, to be consistent with existing enhancers of FANTOM enhancers and known disease-related super-enhancers, scanning human chromosome 22 with our model finds some novel candidate enhancers with supports of histone modification markers of H3K4me1 and H3K27ac.

On the basis of current efforts, we conclude that our method is an accurate and robust model for enhancer identification and we summarize that our work brings some new contributions into the area of enhancer biology:

(1) New contribution to prediction methodology: for the first time, we proposed an innovative method, i.e., selected abelian complexity features together with DNA composition for accurate enhancer identification. In comparison with three state-of-the-art enhancer prediction tools, our method shows greater accuracy and more robust performances on imbalanced datasets.
(2) New contribution to the prediction of novel candidate enhancers: when we applied our trained model to scan human chromosome 22, 7045 genomic regions were identified as enhancers. Some of them are consistent with known FANTOM5 enhancer, and the majority of them are considered as candidate enhancers by the evidences of surrounding histone modification markers including H3K4me1 and H3K27ac. This demonstrates that the predicted enhancers by our scanning approach have the potential to be true enhancers.

Finally, we list two drawbacks or limitations of our model which should be addressed and overcome in future works. The first limitation is the problem of cell-specificity of the enhancer. Our enhancer dataset (Enhancer4589) was constructed by the criteria of “*TPM*_min_ ≥ 0.08“, implying that the enhancers we chose for training are actually ubiquitously active across all tissues and cell lines (termed as “housekeeping enhancers” [51]). A rational inference is that our predicted enhancers are all “housekeeping enhancers”, suggesting that our model has the limitation of predicting cell-specific enhancers. A possible solution is to collect cell-specific enhancers of some particular tissues or cell lines and then construct a prediction model for learning their specific sequence features. Another limitation of current work is the authenticities of the predicted enhancers, which are usually measured by the abilities of driving target gene expression. At this point, further biological experiments such as MPRA are required for a comprehensive evaluation.

## Figures and Tables

**Table 1:** Prediction results of four inputs on dataset Enhancer4589 using a five-fold cross-validation test.

Feature	Dimension	ACC	AUC	MCC	Sens	Spec	GM
SACF484	484	0.910	0.877	0.299	0.914	0.554	0.432
SACF209	209	0.947	0.933	0.579	0.981	0.674	0.715
TNF	256	0.931	0.912	0.491	0.959	0.631	0.639
HACF	465	0.956	0.955	0.627	0.989	0.701	0.755

SACF484: 484 selected abelian complexity features by an upper bound estimation; SACF209: 209 selected abelian complexity features by Wilcoxon-test; TNF: tetra-nucleotide frequency; HACF: hybrid abelian complexity features; ACC: accuracy; AUC: Area Under the ROC Curve; MCC: Matthew’s correlation coefficient; Sens: sensitivity; Spec: specificity.

**Table 2:** Comprehensive comparisons with three existing enhancer prediction methods.

Comparison Targets	Method		ACC	AUC	Sens	Spec	GM
EnhancerFinder [40]	EnhancerFinder		\	0.960	\	\	\
	HACF		0.915	0.962	0.904	0.928	0.916
DEEP [29]	Heart	DEEP	0.822	\	0.802	0.824	0.812
		HACF	0.941	0.919	0.964	0.690	0.734
	Liver	DEEP	0.745	\	0.740	0.755	0.741
		HACF	0.923	0.918	0.935	0.664	0.691
	Brain	DEEP	0.853	\	0.832	0.855	0.843
		HACF	0.940	0.918	0.960	0.698	0.744
iEnhancer-2L [32]	Layer I	iEnhancer-2L	0.769	0.850	0.781	0.759	\
		HACF	0.837	0.907	0.846	0.828	0.838
	Layer II	iEnhancer-2L	0.619	0.660	0.622	0.618	\
		HACF	0.714	0.789	0.713	0.716	0.715

HACF: hybrid abelian complexity features; ‘\’ represents “not provided by original publications”.

## Supplementary Materials

Supplementary materials can be found at https://www.mdpi.com/1422-0067/20/7/1704/s1. Supplementary table S1. The genomic locations of 4589 positives and 45890 negatives of the dataset Enhancer4589; Supplementary table S2. 209 selected abelian complexity features (SACF209); Supplementary table S3. 88 abelian complexity features out of the top 100 important features; Supplementary table S4. Enhancer datasets of kidney, liver and brain from Fantom5 enhancer data; Supplementary table S5. 7045 predicted enhancers by the HACF model.

## Additional Information

Availability and implementation: The R script code of the algorithm implementation of abelian complexity function is freely available at https://github.com/badfroze/abelian-complexity.

## References

[1] Kleftogiannis D., Kalnis P., Bajic V.B. (2015). Progress and challenges in bioinformatics approaches for enhancer identification. Brief. Bioinform..

[2] Shlyueva D., Stampfel G., Stark A. (2014). Transcriptional enhancers: From properties to genome-wide predictions. Nat. Rev. Genet..

[3] Li W., Notani D., Rosenfeld M.G. (2016). Enhancers as non-coding RNA transcription units: Recent insights and future perspectives. Nat. Rev. Genet..

[4] Bulger M., Groudine M. (2011). Functional and mechanistic diversity of distal transcription enhancers. Cell.

[5] Ernst J., Kellis M. (2012). ChromHMM: Automating chromatin-state discovery and characterization. Nat. Methods.

[6] Hoffman M.M., Buske O.J., Wang J., Weng Z., Bilmes J.A., Noble W.S. (2012). Unsupervised pattern discovery in human chromatin structure through genomic segmentation. Nat. Methods.

[7] Fishilevich S., Nudel R., Rappaport N., Hadar R., Plaschkes I., Iny Stein T., Rosen N., Kohn A., Twik M., Safran M. (2017). GeneHancer: Genome-wide integration of enhancers and target genes in GeneCards. Database.

[8] Maurano M.T., Humbert R., Rynes E., Thurman R.E., Haugen E., Wang H., Reynolds A.P., Sandstrom R., Qu H., Brody J. (2012). Systematic localization of common disease-associated variation in regulatory DNA. Science.

[9] Sur I.K., Hallikas O., Vähärautio A., Yan J., Turunen M., Enge M., Taipale M., Karhu A., Aaltonen L.A., Taipale J. (2012). Mice lacking a Myc enhancer that includes human SNP rs6983267 are resistant to intestinal tumors. Science.

[10] Rao S.S., Huntley M.H., Durand N.C., Stamenova E.K., Bochkov I.D., Robinson J.T., Sanborn A.L., Machol I., Omer A.D., Lander E.S. (2014). A 3D map of the human genome at kilobase resolution reveals principles of chromatin looping. Cell.

[11] Diao Y., Fang R., Li B., Meng Z., Yu J., Qiu Y., Lin K.C., Huang H., Liu T., Marina R.J. (2017). A tiling-deletion-based genetic screen for cis-regulatory element identification in mammalian cells. Nat. Methods.

[12] Chapuy B., McKeown M.R., Lin C.Y., Monti S., Roemer M.G., Qi J., Rahl P.B., Sun H.H., Yeda K.T., Doench J.G. (2013). Discovery and characterization of super-enhancer-associated dependencies in diffuse large B cell lymphoma. Cancer Cell.

[13] Lovén J., Hoke H.A., Lin C.Y., Lau A., Orlando D.A., Vakoc C.R., Bradner J.E., Lee T., Young R.A. (2013). Selective inhibition of tumor oncogenes by disruption of super-enhancers. Cell.

[14] Visel A., Minovitsky S., Dubchak I., Pennacchio L.A. (2007). VISTA Enhancer Browser—A database of tissue-specific human enhancers. Nucleic Acids Res..

[15] Visel A., Blow M.J., Li Z., Zhang T., Akiyama J.A., Holt A., Plajzer-Frick I., Shoukry M., Wright C., Chen F. (2009). ChIP-seq accurately predicts tissue-specific activity of enhancers. Nature.

[16] Heintzman N.D., Stuart R.K., Hon G., Fu Y., Ching C.W., Hawkins R.D., Barrera L.O., Van Calcar S., Qu C., Ching K.A. (2007). Distinct and predictive chromatin signatures of transcriptional promoters and enhancers in the human genome. Nat. Genet..

[17] Heintzman N.D., Hon G.C., Hawkins R.D., Kheradpour P., Stark A., Harp L.F., Ye Z., Lee L.K., Stuart R.K., Ching C.W. (2009). Histone modifications at human enhancers reflect global cell-type-specific gene expression. Nature.

[18] Melnikov A., Murugan A., Zhang X., Tesileanu T., Wang L., Rogov P., Feizi S., Gnirke A., Callan C.G., Kinney J.B. (2012). Systematic dissection and optimization of inducible enhancers in human cells using a massively parallel reporter assay. Nat. Biotechnol..

[19] Kwasnieski J.C., Fiore C., Chaudhari H.G., Cohen B.A. (2014). High-throughput functional testing of ENCODE segmentation predictions. Genome Res..

[20] Shen S.Q., Myers C.A., Hughes A.E., Byrne L.C., Flannery J.G., Corbo J.C. (2015). Massively parallel cis-regulatory analysis in the mammalian central nervous system. Genome Res..

[21] Arnold C.D., Gerlach D., Stelzer C., Boryń Ł.M., Rath M., Stark A. (2013). Genome-wide quantitative enhancer activity maps identified by STARR-seq. Science.

[22] Andersson R., Gebhard C., Miguel-Escalada I., Hoof I., Bornholdt J., Boyd M., Chen Y., Zhao X., Schmidl C., Suzuki T. (2014). An atlas of active enhancers across human cell types and tissues. Nature.

[23] Kim T.K., Hemberg M., Gray J.M., Costa A.M., Bear D.M., Wu J., Harmin D.A., Laptewicz M., Barbara-Haley K., Kuersten S. (2010). Widespread transcription at neuronal activity-regulated enhancers. Nature.

[24] Lai F., Gardini A., Zhang A., Shiekhattar R. (2015). Integrator mediates the biogenesis of enhancer RNAs. Nature.

[25] Korkmaz G., Lopes R., Ugalde A.P., Nevedomskaya E., Han R., Myacheva K., Zwart W., Elkon R., Agami R. (2016). Functional genetic screens for enhancer elements in the human genome using CRISPR-Cas9. Nat. Biotechnol..

[26] Yáñez-Cuna J.O., Arnold C.D., Stampfel G., Boryń L.M., Gerlach D., Rath M., Stark A. (2014). Dissection of thousands of cell type-specific enhancers identifies dinucleotide repeat motifs as general enhancer features. Genome Res..

[27] Kvon E.Z., Stampfel G., Yáñez-Cuna J.O., Dickson B.J., Stark A. (2012). HOT regions function as patterned developmental enhancers and have a distinct cis-regulatory signature. Genes Dev..

[28] Catarino R.R., Stark A. (2018). Assessing sufficiency and necessity of enhancer activities for gene expression and the mechanisms of transcription activation. Genes Dev..

[29] Yáñez-Cuna J.O., Kvon E.Z., Stark A. (2013). Deciphering the transcriptional cis-regulatory code. Trends Genet..

[30] Lee D., Karchin R., Beer M.A. (2011). Discriminative prediction of mammalian enhancers from DNA sequence. Genome Res..

[31] Kleftogiannis D., Kalnis P., Bajic V.B. (2014). DEEP: A general computational framework for predicting enhancers. Nucleic Acids Res..

[32] Liu B., Fang L., Long R., Lan X., Chou K.-C. (2015). iEnhancer-2L: A two-layer predictor for identifying enhancers and their strength by pseudo k-tuple nucleotide composition. Bioinformatics.

[33] Yang B., Liu F., Ren C., Ouyang Z., Xie Z., Bo X., Shu W. (2017). BiRen: Predicting enhancers with a deep-learning-based model using the DNA sequence alone. Bioinformatics.

[34] Beer M.A. (2017). Predicting enhancer activity and variant impact using gkm-SVM. Hum. Mutat..

[35] Lothaire M. (2005). Applied Combinatorics on Words.

[36] Koslicki D. (2011). Topological entropy of DNA sequences. Bioinformatics.

[37] Jin S., Tan R., Jiang Q., Xu L., Peng J., Wang Y., Wang Y. (2014). A generalized topological entropy for analyzing the complexity of DNA sequences. PLoS ONE.

[38] Forrest A.R., Kawaji H., Rehli M., Baillie J.K., de Hoon M.J., Haberle V., Lassmann T., Kulakovskiy I.V., Lizio M., Itoh M., FANTOM Consortium and the RIKEN PMI and CLST (DGT) (2014). A promoter-level mammalian expression atlas. Nature.

[39] Erwin G.D., Oksenberg N., Truty R.M., Kostka D., Murphy K.K., Ahituv N., Pollard K.S., Capra J.A. (2014). Integrating diverse datasets improves developmental enhancer prediction. PLoS Comput. Biol..

[40] Richomme G., Saari K., Zamboni L.Q. (2010). Abelian complexity of minimal subshifts. J. Lond. Math. Soc..

[41] Zhang Y., Liu T., Meyer C.A., Eeckhoute J., Johnson D.S., Bernstein B.E., Nusbaum C., Myers R.M., Brown M., Li W. (2008). Model-based analysis of ChIP-Seq (MACS). Genome Biol..

[42] Rajagopal N., Xie W., Li Y., Wagner U., Wang W., Stamatoyannopoulos J., Ernst J., Kellis M., Ren B. (2013). RFECS: A random-forest based algorithm for enhancer identification from chromatin state. PLoS Comput. Biol..

[43] He Y., Gorkin D.U., Dickel D.E., Nery J.R., Castanon R.G., Lee A.Y., Shen Y., Visel A., Pennacchio L.A., Ren B. (2017). Improved regulatory element prediction based on tissue-specific local epigenomic signatures. Proc. Natl. Acad. Sci. USA.

[44] Wang M., Tai C., Wei L. (2018). DeFine: Deep convolutional neural networks accurately quantify intensities of transcription factor-DNA binding and facilitate evaluation of functional non-coding variants. Nucleic Acids Res..

[45] Huang Y., Niu B., Gao Y., Fu L., Li W. (2010). CD-HIT Suite: A web server for clustering and comparing biological sequences. Bioinformatics.

[46] Colosimo A., De Luca A. (2000). Special factors in biological strings. J. Theor. Biol..

[47] Kirillova O.V. (2000). Entropy concepts and DNA investigations. Phys. Lett. A.

[48] Troyanskaya O.G., Arbell O., Koren Y., Landau G.M., Bolshoy A. (2002). Sequence complexity profiles of prokaryotic genomic sequences: A fast algorithm for calculating linguistic complexity. Bioinformatics.

[49] Wu C., Yao S., Li X., Chen C., Hu X. (2017). Genome-Wide Prediction of DNA Methylation Using DNA Composition and Sequence Complexity in Human. Int. J. Mol. Sci..

[50] Allouche J.-P., Shallit J. (2003). Automatic Sequences: Theory, Applications, Generalizations.

[51] Zabidi M.A., Arnold C.D., Schernhuber K., Pagani M., Rath M., Frank O., Stark A. (2014). Enhancer-core-promoter specificity separates developmental and housekeeping gene regulation. Nature.

